# U-CHANGE Project: a multidimensional consensus on how clinicians, patients and caregivers may approach together the new urothelial cancer scenario

**DOI:** 10.3389/fonc.2023.1186103

**Published:** 2023-07-28

**Authors:** Sergio Bracarda, Roberto Iacovelli, Valentina Baldazzi, Paolo Andrea Zucali, Angela Gernone, Giario Natale Conti, Giovanni Pappagallo, Matteo Brunelli, Paolo Bruzzi, Edoardo Fiorini, Laura Magenta, Francesco Diomede, Federico Mereta, Irma D’Aria, Danilo Magliano, Monica Liberatori, Daniela Cantù, Davide Croce, Simone Eandi, Giorgio Lorenzo Colombo, Fulvio Ferrante, Emanuela Omodeo Salè, Andrea Marinozzi, Daniele Lenzi, Francesca Remiddi, Stefano Remiddi

**Affiliations:** ^1^ President of Italian Society of Uro-Oncology, Department of Medical Oncology, Santa Maria Hospital, Terni, Italy; ^2^ Department of Medical Oncology, Fondazione Policlinico Universitario A. Gemelli Istituto di Ricovero e Cura a Carattere Scientifico (IRCCS), Rome, Italy; ^3^ Department of Medical Oncology, Santa Maria Annunziata Hospital, Florence, Italy; ^4^ Department of Biomedical Sciences, Humanitas University, Pieve Emanuele, Milan, Italy; ^5^ Department of Oncology, Istituto di Ricovero e Cura a Carattere Scientifico (IRCCS) Humanitas Research Hospital, Rozzano, Milan, Italy; ^6^ Department of Medical Oncology, Policlinico Universitario Azienda Ospedaliera (A.O), Bari, Italy; ^7^ Italian Society of Uro-Oncology, Bologna, Italy; ^8^ Pathology Unit, Department of Diagnostics and Public Health, University of Verona, Verona, Italy; ^9^ Department of Clinical Epidemiology, National Institute for Cancer Research, Istituto Scientifico Tumori (IST), Genoa, Italy; ^10^ PaLiNUro Association, Milan, Italy; ^11^ Federazione Associazioni Volontariato in Oncologia (F.A.V.O) Federation, Rome, Italy; ^12^ Scientific Journalist, Freelance, Milan, Italy; ^13^ Journalist, Medikea TV, Rome, Italy; ^14^ Journalist, Pharmastar, Milan, Italy; ^15^ Italian Association of Physiotherapists, Milan, Italy; ^16^ Centro di Ricerca sull’Economia e il Management in Sanità e nel Sociale, Libero Istituto Universitario Cattaneo (LIUC) Business School, Castellanza (VA), Turin, Italy; ^17^ Social Innovation EcosystEm Development (SEEd) Medica Publishers, Turin, Italy; ^18^ Studi Analisi Valutazioni Economiche (SAVE) Institute, Milan, Italy; ^19^ Department of Diagnostic and Pharmaceutical Assistance, Unità Operativa Complessa (UOC) Pharmacy, Local Health Unit Azienda Sanitaria Locale (ASL) Frosinone, Frosinone, Italy; ^20^ Department Hospital Pharmacy, IEO, European Institute of Oncology IRCCS, Milan, Italy; ^21^ Clinical Pharmacy, Azienda Ospedaliera Universitaria (AOU) Ospedali Riuniti, Ancona, Italy; ^22^ Medical Department, Azienda Ospedaliera Università, Siena, Italy; ^23^ Medical Writing & Statistics Department, NUME PLUS, Florence, Italy

**Keywords:** advanced urothelial carcinoma, multidimensional consensus, Delphi panel, stakeholders, partnership, molecular tumor board

## Abstract

**Introduction:**

Advanced urothelial carcinoma remains aggressive and very hard to cure, while new treatments will pose a challenge for clinicians and healthcare funding policymakers alike. The U-CHANGE Project aimed to redesign the current model of care for advanced urothelial carcinoma patients to identify limitations (“as is” scenario) and recommend future actions (“to be” scenario).

**Methods:**

Twenty-three subject-matter experts, divided into three groups, analyzed the two scenarios as part of a multidimensional consensus process, developing statements for specific domains of the disease, and a simplified Delphi methodology was used to establish consensus among the experts.

**Results:**

Recommended actions included increasing awareness of the disease, increased training of healthcare professionals, improvement of screening strategies and care pathways, increased support for patients and caregivers and relevant recommendations from molecular tumor boards when comprehensive genomic profiling has to be provided for appropriate patient selection to *ad hoc* targeted therapies.

**Discussion:**

While the innovative new targeted agents have the potential to significantly alter the clinical approach to this highly aggressive disease, the U-CHANGE Project experience shows that the use of these new agents will require a radical shift in the entire model of care, implementing sustainable changes which anticipate the benefits of future treatments, capable of targeting the right patient with the right agent at different stages of the disease.

## 1 Introduction

Urothelial cancer (UC) is the most common histological type of bladder cancer (accounting for 90% of cases) and consists of non-muscle-invasive (approximately two thirds of cases) and muscle-invasive types. Each year, approximately 573,000 new cases of bladder cancer are reported worldwide, with 212,000 deaths. In Europe, approximately 204,000 people were diagnosed with urothelial cancer in 2020, 67,000 of whom died of the disease (1). In Italy, 25,500 people were diagnosed in 2021 with an estimated 6,100 deaths and a 5-year net survival rate of 80% in men and 78% in women (2). 313,600 people in this country are currently living with a diagnosis of bladder cancer (2).

The management of advanced urothelial cancer, as locally pT2, pT3a, pT4 muscle invasive urothelial carcinoma or urothelial carcinoma with metastatic disease, with all its complexities, will certainly become the focus of considerable attention in the immediate future, in part due to new investments in health and social care (such as the Europe’s Beating Cancer Plan, the National Cancer Plan, and the National Recovery and Resilience Plan, a reform and investment package to help Italy recover from the COVID-19 pandemic), and to the imminent arrival on the market of innovative treatment solutions (3, 4). While these treatments promise to improve the survival and quality of life of patients with advanced urothelial cancer, they will also require a new decision-making mindset on the part of clinicians and healthcare administrators at various levels.

The aim of the U-CHANGE Project, where the syllable “U-C” stands for “Urothelial-Cancer”, is to create a shared scenario that combines epidemiological data and diagnostic pathways for a more efficient patient care in the different regional setting, with a perspective of humanization and communication in favor of primary and secondary prevention of urothelial cancer management.

The Project consisted of the examination of the current organizational and clinical management models (including regional models) and the patient journey, creating two “snapshots”: one “as is” scenario of the current situation, and one “to be” scenario, to be implemented in the immediate future.

While multidisciplinary consensus processes have been used several times in oncology, they have always been held among peers (5). The U-CHANGE Project chose instead to adopt a multidimensional simplified Delphi-consensus approach (6–8), setting itself the ambitious and unprecedented objective of putting on the same level the various figures who deal with the advanced urothelial carcinoma patient at the various stages of their journey: clinicians, patient associations, caregivers, physiotherapists, nurses, healthcare journalists, hospital pharmacists, directors of healthcare trusts and hospitals, as well as local, regional and national healthcare economists.

## 2 Materials and methods

### 2.1 Expert panel

The U-CHANGE expert panel consisted of twenty-three experts who were invited to participate in the two in-person consensus meetings. The experts were divided into three groups: Clinicians (eight), Patients (eight) and Institutions (seven) and were deemed to be representative of the professional categories which directly influence patient care in Italy.

The Clinicians group was composed by: five oncologists (SB, RI, VB, PAZ, AG), one urologist (GNC), one methodologist (GP) and one epidemiologist (PB). In addition, one anatomopathologist (MB) has been involved in the revision of the manuscript. The Patients group was composed by: three representatives of patient’s associations (EF, LM, FD), three scientific journalists (FM, ID, DM), one nurse (ML) and one physiotherapist (DCA). The Institutions group was composed by: three health economists (DCR, SE, GLC), three hospital pharmacists (FF, EOS, AM) and one local health trust director (DL).

### 2.2 Systematic review of literature and development of the statements

**Figure 1:**
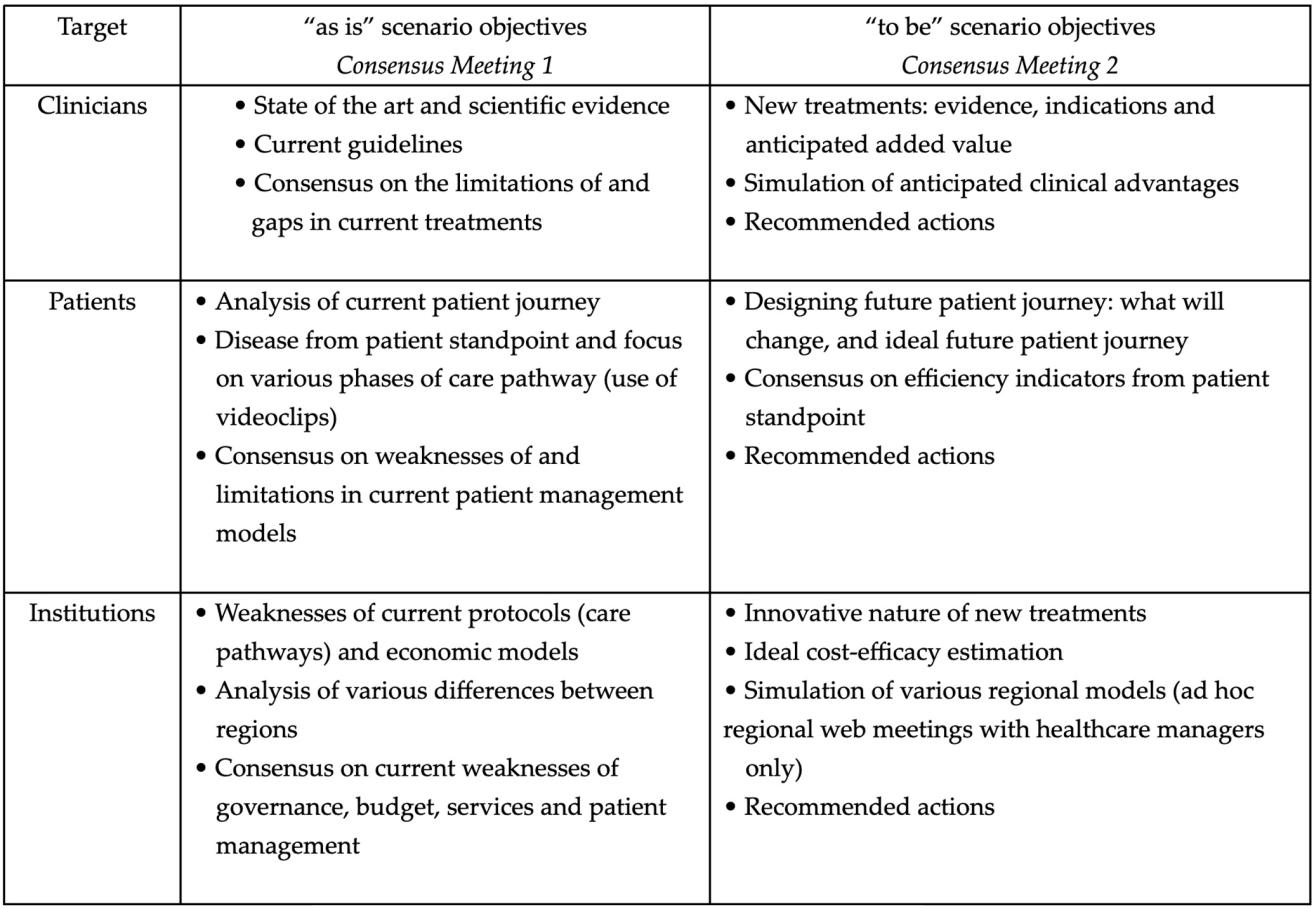
Development of the U-CHANGE Project.

As shown in **Figure 1**, the U-CHANGE Project was designed and developed in the form of three dimensions (Clinicians, Patients and Institutions) and two scenarios (“as is” and “to be”). Prior of each meeting, each expert was sent a document containing the possible statements for each domain which would be discussed and voted on, each supported by the most recent published scientific evidence.

### 2.3 Voting and degree of agreement

The draft of the document containing the various statements had been emailed to all twenty-three panel members prior to the first consensus meeting, together with an explanation of the project objectives and instructions for the group work in each dimension. At each meeting, following the discussion by each group, the definitive statements were presented and then voted on by all participants. Participants were asked to rate their agreement with each recommendation on a 5-point Likert scale (ranging from “strongly agree” to “strongly disagree”), and an agreement threshold of 75% to reach consensus (calculated as sum of agree and strongly agree) was chosen based on the method described by Loblaw et al. (9).

### 2.4 Preparation of consensus document and sharing with all experts

**Figure 2:**
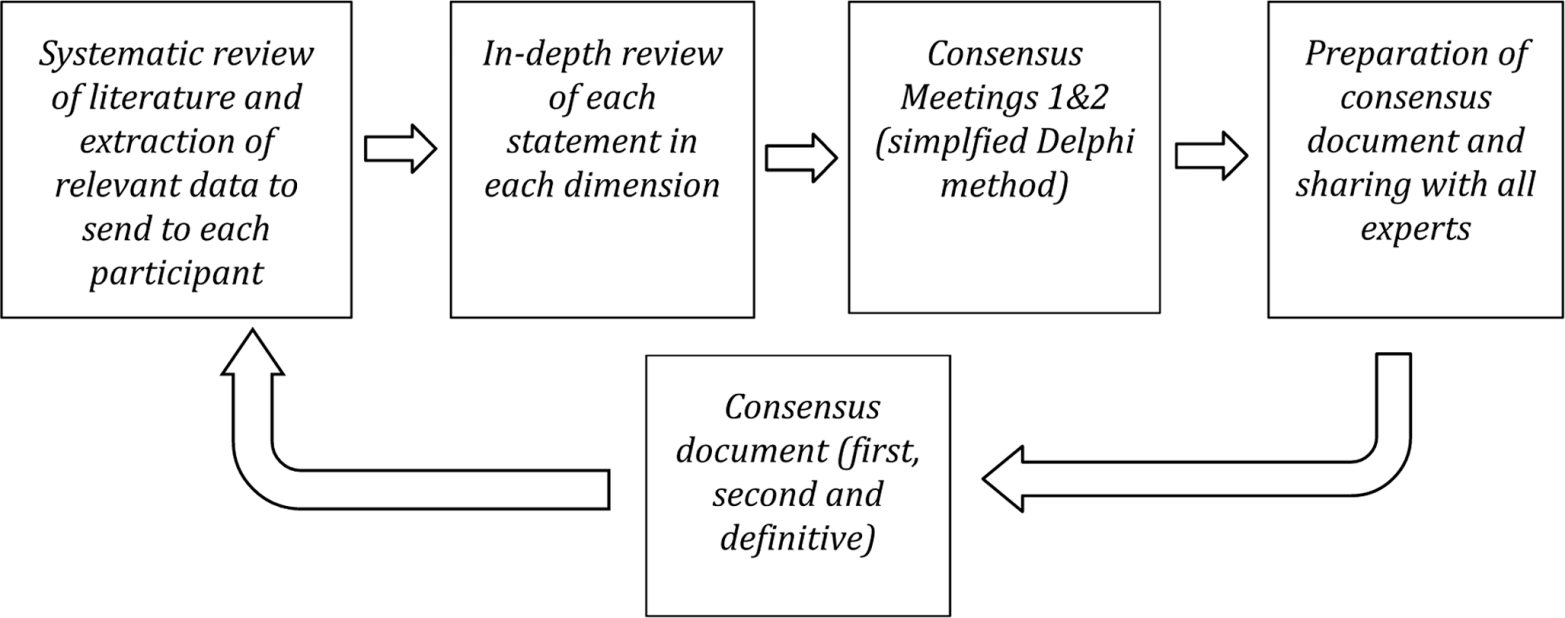
Flow diagram of U-CHANGE Project.

Following the first consensus meeting on the “as is” scenario, the first report discussed with the chairmen of the three groups was sent out, and the statements to be developed for the “to be” scenario (second consensus meeting) were identified. Once this process had been repeated with the second scenario, the final consensus document was drawn up (as shown in **Figure 2**).

### 2.5 Ethics approval

This study consisted of a report of expert opinions that are from the authors, who all have given consent to the use of their information and opinion for this manuscript. No patient data were collected, so no ethical approval was required to perform this study.

## 3 Results

For the “as is” scenario, participants voted on a total of sixteen statements (six for the Clinicians dimension, five for the Patients dimension, and five for the Institutions dimension). The degree of agreement, following the first round of voting, was over 75% for fifteen statements. Only one statement (no. 9) did not reach consensus due to an insufficient level of agreement, and was reformulated and put to a second round of voting (no. 9b). One statement (no. 11) failed to reach consensus following in-depth discussion and was withdrawn from voting.

For the “to be” scenario, participants voted on a total of fourteen statements (five for the Clinicians dimension, four for the Patients dimension, and five for the Institutions dimension), and the degree of agreement exceeded 75% for all statements following the first round of voting.

This section gives the results of the voting of the entire expert panel for each dimension (Clinicians, Patients and Institutions) on each statement of the “as is” scenario compared with the “to be” scenario and comments reported by all the expert panel.

### 3.1 Clinicians dimension: “as is” and “to be” scenarios compared

This section gives the results of the voting by the entire panel on each statement, summarized in **Table 1**.

**Table 1:** Clinicians dimension.

DOMAIN	SCENARIO	No.	*STATEMENT*	Strongly agree + agree (%)	Strongly agree (%)	Agree (%)	Neither agree nor disagree (%)	Strongly disagree + disagree (%)
Survival	*AS IS*	1	Despite the treatment options available, locally advanced/metastatic urothelial carcinoma remains a disease characterized by a high probability of progression and therefore difficult to cure (1, 2, 10–14)	**100%**	75%	25%	0%	0%
Awareness	*TO BE*	17	Healthcare institutions should run information campaigns on the disease (signs, symptoms, risk factors) to reduce the number of late-stage diagnoses, with separate campaigns for general physicians, specialists and the general public (14–20)	**100%**	62%	38%	0%	0%
Diagnosis	*AS IS*	2	There is a lack of scientific evidence to support screening programs (in asymptomatic subjects) for the general public and in groups at high risk for urothelial carcinoma (2, 12, 14–17, 21–23)	**100%**	65%	35%	0%	0%
	*AS IS*	3	There is a need to increase awareness of the disease among the general public and the medical community to allow more rapid diagnosis (12, 17–19)	**100%**	80%	20%	0%	0%
	*TO BE*	18	Scientific research should include activities aimed at identifying new, non-invasive diagnostic strategies (biomarkers) to allow a more rapid diagnosis of urothelial cancer (12, 15 17, 18, 21–27)	**100%**	62%	38%	0%	0%
Treatment	*AS IS*	4	Despite the recent improvements in treatment options for locally advanced/metastatic urothelial cancer, there should be a focus on increasing personalized treatment for the disease (12, 22)	**95%**	65%	30%	5%	0%
	*TO BE*	19	The increasing availability of targeted therapies will result in more specific treatment pathways for individual patients, and improve survival and quality of life (12, 22, 28–38)	**100%**	62%	38%	0%	0%
Multidisciplinary team	*AS IS*	5	Early management of the urothelial carcinoma patient by a multidisciplinary team is crucial to guarantee access to the most appropriate treatment options (17, 38–42)	**100%**	55%	45%	0%	0%
	*TO BE*	20	Regional health services should create multidisciplinary teams to manage the entire patient journey from diagnosis onwards, with care pathways designed and shared by all team members (17, 38–44)	**95%**	71%	24%	5%	0%
Community healthcare services	*AS IS*	6	The considerable differences in the management and continuity of care for patients with urothelial cancer throughout Italy should be progressively reduced (14, 45)	**95%**	75%	25%	5%	0%
	*TO BE*	21	All regional health services should optimize the care pathway for patients with urothelial cancer, starting with an assessment of existing practices to identify the most appropriate organizational models (definition of care pathways) (14, 45–47)	**100%**	62%	38%	0%	0%

“as is” and “to be” scenarios.Percentages in bold indicate where strongly agree+agree (%) overpass 75% cut-off point.

#### 3.1.1 Clinicians: comments relative to the clinicians dimension statements

The following section gives the most relevant comments which emerged from the discussion with regard to each statement, grouped by domain.

##### 3.1.1.1 Domain: survival

“As is” scenario - Despite the treatment options available, locally advanced/metastatic urothelial carcinoma remains a disease characterized by a high probability of progression and therefore difficult to cure.

The current treatment scenario is constantly evolving. Despite the contrasting data on efficacy and survival, the latest updates on the results of immune checkpoint inhibitor registration studies were given during the discussion. The data currently available, not considering treatments set to become available in the near future, show that locally advanced/metastatic urothelial cancer remains a disease with a high probability of progression, and that the disease remains difficult to cure, above and beyond the availability of second or subsequent lines of treatment and the response rate to these.

“To be” scenario – In this case, because of the uncertainty of the survival data expected in the future, Clinicians decided not to vote this domain.

##### 3.1.1.2 Domain: awareness

“As is” scenario – Because the level of awareness in the population regarding this disease is considered very low, Clinicians decided not to vote this domain and evaluate “To be” scenario only.

“To be” scenario – Healthcare institutions should run information campaigns on the disease (signs, symptoms, risk factors) to reduce the number of late stage diagnoses, with separate campaigns for general practitioners, specialists and the general public.

There is no scientific evidence to support screening campaigns for urothelial cancer with the goal of early diagnosis among asymptomatic subjects or those in high-risk groups. The discussion highlighted a two-fold need to raise awareness of the disease: firstly to educate the general public about the need to promptly consult their general practitioner in the event of suspicious symptoms, and secondly to prompt general practitioner to order diagnostic tests for patients with risk factors at the onset of signs and symptoms.

It is not unusual, in fact, for female patients especially, to be prescribed lengthy courses of antibiotics for symptoms attributed to recurrent cystitis, and a first-line diagnostic approach in patients with macrohematuria and a history of recurrent cystitis should consist of non-invasive tests such as urine cytology and bladder ultrasound. Further resources for increasing awareness of the disease include: campaigns to promote a healthy lifestyle; fostering alliances between general practictioner, community healthcare services and regional cancer networks; increasing awareness of symptoms and risk factors, and the provision of multidisciplinary training opportunities (run by scientific societies if possible) for urologists, oncologists and radiotherapists, as well as for general physicians. These health campaigns could be run on innovative and popular platforms (podcasts, short online interviews, leaflets, social media campaigns, etc.), using material prepared by a multidisciplinary scientific committee, taking care to avoid misunderstandings and misinformation.

##### 3.1.1.3 Domain: diagnosis

“As is” scenario - There is a lack of scientific evidence to support screening programs (in asymptomatic subjects) for the general public and in groups at high risk for urothelial carcinoma.

The aim of a screening program is to detect the disease at its early stages in members of the general public or people with specific risk factors who have no symptoms. Efforts to introduce screening programs for urothelial carcinoma are currently hampered by a lack of prerequisites and scientific evidence to support the existing first level tests.

“As is” scenario - There is a need to increase awareness of the disease among the general public and the medical community to allow more rapid diagnosis.

Awareness of urothelial cancer is poor, not only among the general public but also in the medical community, often delaying recognition of the signs and symptoms of the disease, resulting in significantly longer diagnosis times. Achieving a greater awareness of the disease and its signs and symptoms to optimize the current care pathways and speed up diagnostic times should therefore be a priority.

“To be” scenario – Scientific research should include activities aimed at identifying new, non-invasive diagnostic strategies (biomarkers) to allow a more rapid diagnosis of urothelial cancer.

The current lack of non invasive diagnostic tests is a tremendous hindrance to the rapid diagnosis of urothelial cancer. Increasing those avenues of scientific research aimed at identifying new biomarkers (such as imaging or blood tests, whether invasive or non invasive) able to detect the disease as early as possible, and/or assess the efficacy of treatment in selected population groups, is therefore of crucial importance.

##### 3.1.1.4 Domain: treatment

“As is” scenario - Despite the recent improvements in treatment options for locally advanced/metastatic urothelial cancer, there should be a focus on increasing personalized treatment for the disease.

The treatment options currently available for locally advanced/metastatic urothelial cancer consist of an already established neoadjuvant treatment, and an adjuvant treatment which has undergone recent important changes, with a first line of treatment, subsequent maintenance line and second line of treatment. Two further drugs for a specific group of patients will also soon be available. Although this treatment scenario is in constant evolution, with a recent increase in effective treatment options, the treatment lines subsequent to the first remain unsatisfactory. Despite the recent improvements in terms of survival and treatment options available, more needs to be done to personalize the current treatment options to render them satisfactory in terms of efficacy. At a time when promising new treatment prospects are on the horizon, optimizing the treatment sequence and identifying eligible patients is more important than ever.

“To be” scenario – The increasing availability of targeted therapies will result in more specific treatment pathways for individual patients, and improve survival and quality of life.

The new treatment options, when available, will increase survival yet further, with a lower incidence of side effects and an improved quality of life. While truly personalized treatment is still a long way off due to the limited choice of molecular targeted therapies (growing but still extremely narrow), precision medicine drugs and techniques such as genetic analysis, next generation sequencing and biomoleculars will permit clinicians to gather more and more information on tumor characteristics, biological heterogeneity, the variability in prognoses and response to treatment, thus allowing improved personalization of the treatment algorithm to each individual patient. Clinicians must be careful not to lose sight of the person/patient as a whole, with their own specific comorbidities, in-dividual characteristics, expectations and everything which goes to make them the unique individual that they are, exploiting all the potential of precision medicine during diagnosis, treatment and follow-up. A greater availability of molecular targeted therapies will therefore allow more appropriate and more specific care pathways, with a wider choice of treatments compared to now. Better tailoring care pathways to the needs of patients will hopefully improve the relationship between desirable and undesirable effects of treatment, improving patients’ quality of life.

##### 3.1.1.5 Domain: multidisciplinary team

“As is” scenario - Early management of patients with urothelial carcinoma by a multidisciplinary team (MDT) is crucial to guarantee access to the most appropriate treatment options.

MDTs are not currently a feature of all healthcare services, while patient management in others is slow and/or inappropriate. To ensure an optimal care pathway, therefore, it is vital that those healthcare services devoid of MDT direct the patient with bladder cancer to facilities where they can be properly managed from the outset.

“To be” scenario – Regional health services should create MDTs to manage the entire patient journey from diagnosis onwards, with care pathways designed and shared by all team members.

The MDT should consist of professionals from a variety of health professions. To ensure that this happens in practice and not just on paper, it is crucial to define the minimum requirements for the creation of both the core team of professionals, and the non core team of members who join as the need arises (e.g. pharmacologist, molecular biologist, geneticist, geriatric oncologist, oncology psychologist, nephrologist, etc.). Internal audits and periodic staff meetings should also be scheduled to ensure that the correct procedures are being used and that the continuing professional education goals of the group are being met (at least one training event per year).

##### 3.1.1.6 Domain: community healthcare services

“As is” scenario - The considerable differences in the management and continuity of care for patients with urothelial cancer throughout Italy should be progressively reduced.

Italy’s regional cancer networks differ widely throughout the country, and their work is often ineffective, one reason for the regional differences in patient management.

“To be” scenario – All regional health services should optimize the care pathway for patients with urothelial cancer, starting with an assessment of existing practices to identify the most appropriate organizational models (definition of care pathways).

Defining a one-size-fits-all organizational model is not feasible, therefore a thorough analysis of the current situation in each individual area should be performed to identify the most appropriate organizational model, vital for guaranteeing that patient management begins as soon as possible. Once the most appropriate model has been identified, the regional health services should optimize the care network in such as way as to maximize the integration between hospitals, hospital networks and community services, *via* the adoption of guidelines for the integrated management of the care pathways.

### 3.2 Patients dimension: “as is” and “to be” scenarios compared

This section gives the results of the voting by the entire panel on each statement, summarized in **Table 2**.

**Table 2:** Patients dimension.

DOMAIN	SCENARIO	No.	*STATEMENT*	Strongly agree + agree (%)	Strongly agree (%)	Agree (%)	Neither agree nor disagree (%)	Strongly disagree + disagree (%)
Awareness	*AS IS*	7	Training courses currently devote few resources to the signs, symptoms and dysfunctions associated with urothelial cancer. There is insufficient knowledge of the disease among the general public, general physicians and gynecologists, and not enough being done to raise awareness (17, 19)	**100%**	50%	50%	0%	0%
	*TO BE*	22	Scientific societies and patient associations should involve the institutions and other healthcare professionals in campaigns on the importance of an early diagnosis, risk factors and treatment opportunities. These campaigns should target different population groups and use a variety of communication tools (17, 45)	**100%**	43%	57%	0%	0%
Holistic approach to caregiver	*AS IS*	8	Patient caregivers (spouses/partners/family members), are often poorly informed, trained and supported (psychological support and training in how to care for patients) during the various stages of the disease (46, 48, 49)	**95%**	65%	30%	5%	0%
	*TO BE*	24	Scientific societies and patient associations should support caregivers in the form of specific strategies and in-person meetings to provide concrete support (psychological, pharmacological, educational and logistical, in collaboration with the relative treatment facilities) to allow proper management of the disease at its various stages (46, 48, 49)	**90%**	38%	52%	10%	0%
Quality of life	*AS IS*	9	Caregivers should be offered support in those areas which impact the quality of life of patients with a diagnosis of advanced urothelial cancer, especially those who have undergone radical cystectomy (50, 51)	58%	48%	10%	32%	10%
	*AS IS*	9b	In patients with advanced urothelial cancer, especially those who have undergone radical cystectomy, the physical, psychological and social aspects which hinder the patient’s potential to adapt to the changes brought on by the disease have a negative impact on the patient, and do not receive enough attention (50, 51)	**100%**	63%	30%	0%	0%
Holistic approach to patient	*AS IS*	10	All too rarely are patients offered services such as psychological screening, support programs, nutritional counseling or rehabilitative physiotherapy during the times of greatest vulnerability such as diagnosis, the beginning or end of treatment, relapse or progression of the disease (17, 48, 50, 52, 53)	**95%**	50%	45%	5%	0%
	*TO BE*	23	By adopting a common language, scientific societies and patient associations should establish criteria which define best practices (psychological screening, support programs, nutritional counseling or rehabilitative physiotherapy at all stages of the patient journey), with the support of telemedicine where possible (17, 48, 50, 52, 53)	**100%**	43%	57%	0%	0%
Role of patient associations	*TO BE*	25	Scientific societies and other stakeholders should recognize the role of patient associations as a reference point for the patient and the caregiver, with agreed and defined limitations, a role which is complementary to treatment facilities, offering tools and services such as awareness raising, self-help groups and educational material (39, 46)	**100%**	57%	43%	0%	0%

”as is” and “to be” scenarios.Percentages in bold indicate where strongly agree+agree (%) overpass 75% cut-off point.

#### 3.2.1 Patients: comments related to the Patients dimension statements

The following section gives the most relevant comments which emerged from the discussion with regard to each statement, grouped by domain.

##### 3.2.1.1 Domain: awareness

“As is” scenario - Training courses currently devote few resources to the signs, symptoms and dysfunctions associated with urothelial cancer. There is insufficient knowledge and awareness of the disease among the general public, general practitioners (GPs) and gynecologists.

Participants discussed the poor awareness of urothelial cancer among patients and GPs. The main problems which emerge for this condition, complex and neglected by doctors, lie in the scanty health promotion of this particular cancer, use of terminology difficult for patients to understand, and the little importance given to the training of GPs in the field of urology and in perineal diseases more specifically. Patient experience shows that GPs often do not associate signs such as macrohematuria with urothelial cancer, often leading to the prescription of unsuitable treatments and patients being sent to the wrong specialists (e.g. men with pelvic floor dysfunction sent to a psychologist, or women with hematuria sent to a gynecologist or repeatedly treated for recurrent cystitis).

“To be” scenario – Scientific societies and patient associations should involve the institutions and other healthcare professionals in campaigns on the importance of an early diagnosis, risk factors and treatment opportunities. These campaigns should target different population groups and use a variety of communication tools.

To be effective, health campaigns require a collaboration between the scientific societies and patient associations, and should involve the institutions and various healthcare professionals involved in the care pathway. The discussion on the involvement of other healthcare professionals, such as geriatrists, gynecologists, nurses, physiotherapists and GPs, showed that these professionals should be properly involved and trained, *via* the creation and distribution of health promotion material where necessary. Participants also discussed the need to bridge the current educational gap where the multidisciplinary approach is concerned to bring about a cultural change from the bottom up.

##### 3.2.1.2 Domain: holistic approach to the caregiver

“As is” scenario - Patient caregivers (spouses/partners/family members), are often poorly informed, trained and supported (psychological support and training in how to care for patients) during the various stages of the disease.

Discussion of the current care model offered to patient caregivers showed a number of critical areas arising from the scarce resources devoted to the cancer plan, and from the patchy and fragmented nature of health and social care throughout the country. Very often caregivers are unprepared, poorly informed, and are offered no support in caring for the patient at home after surgery. Little attention is often paid to the patient’s overall care process, from diagnosis to their return home, thus contributing to a failure to the meet the needs of the caregiver, often overburdened by the economic aspects of the care process. The lack of attention to these aspects often triggers negative emotional experiences and feelings of disorientation, distrust, neglect, distress and loneliness. Patient associations provide assistance with these issues and provide a reference point for caregivers and the patients themselves.

“To be” scenario - Scientific societies and patient associations should support caregivers in the form of specific strategies and in person meetings to provide concrete support (psychological, pharmacological, educational and logistical, in collaboration with the relative treatment facilities) to allow proper management of the disease at its various stages.

To promote caregiver involvement in patient management, caregivers should be offered training in the everyday issues of caring for patients with the disease, social and psychological support (meeting the needs of the patient as well as their own), as well as peer support, considered to be the best source of emotional support and exchange of good practices. The informal caregiver is a fundamental element in patient care, not only during the final stages of the disease (such as caring for the patient at home following surgery), but at all stages of the patient journey, therefore they should be provided with the same information as the patient, and be supported throughout the entire course of the disease.

##### 3.2.1.3 Domain: quality of life

“As is” scenario - In patients with advanced urothelial cancer, especially those who have undergone radical cystectomy, and those patients who after surgery will subsequently receive appropriate oncological medical therapies, the physical, psychological and social aspects which hinder the patient’s potential to adapt to the changes brought on by the disease have a negative impact on the patient, and do not receive enough attention.

Patients increasingly report feeling abandoned after surgery, with a negative impact on their quality of life, and feel that their relationship with their doctor is not always adequate at the times of greatest vulnerability. Another frequent complaint is of being poorly informed of the specialist services available, requiring them to seek physiotherapy and psychological, nutritional and nursing support by themselves.

“To be” scenario – For this domain, the discussion was included in the in the “Holistic approach to the patient” domain

##### 3.2.1.4 Domain: holistic approach to the patient

“As is” scenario -All too rarely are patients offered services such as psychological screening, support programs, nutritional counseling or rehabilitative physiotherapy during the times of greatest vulnerability such as diagnosis, the beginning or end of treatment, relapse or progression of the disease.

In addition to specific and subjective needs, participants discussed the importance of ensuring patients are offered support services such as psychotherapy, rehabilitation, nutrition advice and physiotherapy. Particular attention was paid to sexual health, an aspect all too often overlooked. It is therefore crucial to identify when the patient is at their most vulnerable to ensure that any psychological, physical or social assessments are carried out at the most appropriate time.

“To be” scenario - By adopting a common language, scientific societies and patient associations should establish criteria which define best practices (psychological screening, support programs, nutritional counseling or rehabilitative physiotherapy at all stages of the patient journey), with the support of telemedicine where possible.

Participants considered scientific societies and patient associations to be in the best position to define good patient support practices (guidelines and care pathways). To promote a greater awareness of the multidisciplinary model among healthcare workers and to encourage patient engagement, the adoption of a common language which encourages a collaborative approach to defining effective practices appears to be useful. Increasing the patient’s awareness of their disease, its course and the treatment options available, the potential consequences of deviating from the agreed treatment plan, and the support services available were all identified as promoting the therapeutic alliance and patient engagement, preventing the patient from entering a phase known as “blackout” (54), characterized by sensations of paralysis, sadness and anger.

##### 3.2.1.5 Domain: role of patient associations

“As is” scenario – Because the current role of patient associations for this disease was considered not relevant, this table decided not to vote this domain and evaluate “To be” scenario only.

“To be” scenario - Scientific societies and other stakeholders should recognize the role of patient associations as a reference point for the patient and the caregiver, with agreed and defined limitations, a role which is complementary to treatment facilities, offering tools and services such as awareness raising, self-help groups and educational material.

The patient associations feel that their role, together with that of the scientific societies and institutions, should be redefined to reflect their value as qualified and expert figures in the patient care process, and that their involvement be reviewed with a view to gaining formal recognition of this role. The aim is to develop information campaigns, awareness raising initiatives and health promotion materials which meet the specific needs of patients and their caregivers, with the objective of raising the profile of urothelial cancer, putting it on the same footing as other cancers which receive more attention from the institutions and the scientific community.

### 3.3 Institutions dimension: “as is” and “to be” scenarios compared

This section gives the results of the voting by the entire panel on each statement, summarized in **Table 3**.

**Table 3:** Institutions dimension.

DOMAIN	SCENARIO	No.	*STATEMENT*	Strongly agree + agree (%)	Strongly agree (%)	Agree (%)	Neither agree nor disagree (%)	Strongly disagree + disagree (%)
Awareness	*AS IS*	11	Health campaigns aimed at the general public are inefficient: it is important to be aware of this, develop targeted strategies, identify the potential for improvement and define the relative budget (12, 17, 24, 25)			Withdrawn from voting		
Diagnosis	*AS IS*	12	Care pathways are few and concentrated in a few areas. There are also significant differences in monitoring indicators and in how often these pathways should be updated (50, 55–62)	**88%**	63%	25%	6%	6%
	*AS IS*	13	There are no specific programs or resources for the healthcare facilities and professionals involved in the care pathways (including general physicians) to help with the creation or implementation of patient support pathways (not even at home or remotely) (17, 45, 46)	**94%**	47%	47%	0%	6%
	*TO BE*	26	Promote specific regional care pathways to standardize the management, treatment and follow-up of patients with urothelial cancer across the country, suggesting monitoring indicators which are sufficiently uniform, and a standardized timeframe for updates to these pathways (24, 55–63)	**100%**	48%	52%	0%	0%
	*TO BE*	27	To best meet the patient’s needs throughout all stages of their illness, institutions, patient associations and scientific societies should promote specific training programs tailored to all healthcare professionals involved in the care pathways (including general physicians and gynecologists), ensuring these programs are also based on the current reorganization of community health services (creation of community health centers) (17, 45, 48)	**90%**	52%	38%	10%	0%
Treatment	*AS IS*	14	There are areas of the current patient management cost model (targeted assessments of surgical, medical and nursing needs) where the care pathway can be improved and streamlined at all stages of the disease (17, 46)	**94%**	22%	72%	6%	0%
Multidisciplinary team	*AS IS*	15	MDT treatment has been shown to improve the appropriacy of diagnostic and treatment procedures, but is not always offered to all patients at high risk of progression (17, 38–43)	**100%**	61%	39%	0%	0%
	*TO BE*	29	Regional health service trusts should review the internal governance of their treatment units and healthcare facilities to promote the creation of MDTs, fundamental for the management of patients with urothelial cancer (17, 38–42, 64)	**100%**	57%	43%	0%	0%
Efficiency of expenditure	*AS IS*	16	While expenditure is low in terms of total cost (5% of expenditure for all cancers), high costs are generated per patient and for the national health service due to subsequent relapses. Total expenditure for the advanced forms tends to decrease due to the reduced number of patients to treat, but with an increase in average cost per patient given medical treatment alone (14, 17, 64–66)	**100%**	26%	74%	0%	0%
Increase efficiency of patient management	*TO BE*	28	Assess the possible areas where current patient management models could be improved, and propose innovative solutions to minimize any gaps (e.g. regional cancer networks with different models, training, use of telemedicine) (45, 47, 54)	**100%**	38%	62%	0%	0%
Budget increase	*TO BE*	30	In light of the future improvements in patient survival and quality of life (new treatments, creation of care pathways), institutions should plan to increase/redistribute the budget allocated to the management of this disease, taking the possible savings generated by an improvement in the efficiency of the patient management model into account (14, 17, 20, 65, 66)	**100%**	62%	38%	0%	0%

“as is” and “to be” scenarios.Percentages in bold indicate where strongly agree+agree (%) overpass 75% cut-off point.

#### 3.3.1 Institutions: comments related to the institutions dimension statements

The following section gives the most relevant comments which emerged from the discussion with regard to each statement, grouped by domain.

##### 3.3.1.1 Domain: awareness

“As is” scenario - Health campaigns aimed at the general public are inefficient: it is important to be aware of this, develop targeted strategies, identify the potential for improvement and define the relative budget.

Universal screening for the disease in asymptomatic individuals is clinically problematic due to the poor specificity and sensitivity of non invasive tests, and the high costs and invasive nature of cystoscopy, the gold standard test, one of the diagnostic tests most of them prescribed along with complete urine tests and urinary tract ultrasound. There is also no standard protocol which defines the timeline for diagnostic tests.

This statement was therefore withdrawn from the voting process.

##### 3.3.1.2 Domain: diagnosis

“As is” scenario - Care pathways are few and concentrated in a few areas. There are also significant differences in terms of monitoring indicators and how often these pathways should be updated.

An analysis of the care pathways available from 2014 to nowadays, showed the most complete to be those of the Veneto and Campania regions, created with the support of the regional cancer networks. Quantification of the indicators in these pathways is often insufficient (see appropriate monitoring indicators in the Veneto care pathway), due in part to the lack of scientific evidence (with a cut-off of twenty radical cystectomies/year, only 18-20% of facilities meet this threshold). The main limitation of the indicators in the Campania region care pathway lies in the fact that the only indicator is the maximum time within which each service should be performed.

Finally, participants highlighted the lack of a hospital pharmacist in the care pathway, and the need to improve the definition of MDT (palliative care is not taken into consideration).

“As is” scenario - There are no specific programs or resources for the healthcare facilities and professionals involved in the care pathways (including general practitioners) to help with the creation or implementation of patient support pathways (not even at home or remotely).

The latest data on the regional cancer networks collected by the Italian national agency for regional healthcare services (Agenas) shows a national map with ten different management, organizational and reference models for oncology (June 2021). The prevalent models are the comprehensive cancer center and the hub & spoke, while the type of healthcare workers involved varies widely. In addition, general practitioners are often not trained to recognize the early signs and symptoms of bladder cancer, especially in women, factors which risk increasing the incidence of the disease and delaying diagnosis.

The economic resources dedicated to the disease are currently used for diagnostic confirmation, long term patient monitoring, and to provide the specialist pathways with the necessary facilities and personnel. Home drug delivery is currently restricted to oral treatments for palliative and ancillary care, but much depends on local facilities.

“To be” scenario - Promote specific regional care pathways to standardize the management, treatment and follow-up of patients with urothelial cancer across the country, suggesting monitoring indicators which are sufficiently uniform, and a standardized timeframe for updates to these pathways.

Scientific societies and institutions should promote the adoption of a common language and a standard methodology for the creation of a regional care pathway in the various regions, specifying the minimum indicators (outcome, quality and appropriacy, including the critical aspects relative to diagnostic imaging), while respecting the diversity of the existing care pathways at individual healthcare facilities. Each region can then formulate each objective based on their own healthcare facilities and available resources.

“To be” scenario - To best meet the patient’s needs throughout all stages of their illness, institutions, patient associations and scientific societies should promote specific training programs tailored to all healthcare professionals involved in the care pathways (including general physicians and gynecologists), ensuring these programs are also based on the current reorganization of community health services (creation of community health centers).

Participants highlighted the need to develop tailored training programs to help the various healthcare professionals identify the early signs of the disease. The new community health centers will have a urology specialist (for the treatment of prostate-related issues), thus providing a resource for bladder cancer. Scheduling audits and reviews of the care pathways may also be useful.

##### 3.3.1.3 Domain: treatment

“As is” scenario - There are areas of the current patient management cost model (targeted assessments of surgical, medical and nursing needs) where the care pathway can be improved and streamlined at all stages of the disease.

The current treatment cost model is mainly based on drug treatments but should be improved to incorporate the cost of the entire care pathway, including hospital and patient care, follow ups, and measures to lessen the social and psychological impact of the disease and improve quality of life.

“To be” scenario – This domain has been reformulated and splitted into the two last domains

##### 3.3.1.4 Domain: multidisciplinary team

“As is” scenario - MDT treatment has been shown to improve the appropriacy of diagnostic and treatment procedures, but it is not always offered to all patients at high risk of progression.

MDT analysis showed teams to be drawn from twelve different healthcare professions: urologist, medical oncologist, radiotherapist, pathologist, radiologist, oncology psychologist, palliative care specialist, anesthetist, nurse, case manager, stoma nurse and rehabilitation technician. Not all teams contained members from all twelve professions.

The possible objectives of an MDT were discussed, and included continuing professional development of its members, use of the best treatment option, and management of the entire patient journey (from diagnosis to follow-up).

“To be” scenario - Regional health service trusts should review the internal governance of their treatment units and healthcare facilities to promote the creation of MDTs, fundamental for the management of patients with urothelial cancer.

In view of the upcoming funding provisions, such as the 20 million Euro fund allocated by the regions to provide psychological assistance to the most vulnerable sectors of the population, with priority given to cancer patients, participants stressed the importance of the MDT as a fundamental and characteristic element of the care pathway for this type of cancer.

##### 3.3.1.5 Domain: improving efficiency of expenditure

“As is” scenario - While expenditure is low in terms of total cost (5% of expenditure for all cancers), high costs are generated per patient and for the national health service due to subsequent relapses. Total expenditure for the advanced forms tends to decrease due to the reduced number of patients to treat, but with an increase in average cost per patient given medical treatment alone.

While the management of bladder cancer weighs heavily on the healthcare budget due to the high percentage of relapses, intensive surveillance strategies, and the high cost of treatments, analysis of the direct costs of the disease shows the new first and second line chemotherapy drugs (more costly than current treatments) to be inferior to the high costs of radiotherapy and radical cystectomy.

“To be” scenario – This domain has been reformulated and split into the two last domains

##### 3.3.1.6 Domain: Streamlining patient management

“As is” – See explanation above (3.3.1.3)

“To be” scenario - Assess the possible areas where current patient management models could be improved, and propose innovative solutions to minimize any gaps (e.g. regional cancer networks with different models, training, use of telemedicine).

Analysis by Agenas of the work of the regional cancer networks shows a more positive picture than that found in reality, especially given the lack of data on bladder cancer (e.g. regional registries for this type of cancer). Less than half of the regions surveyed offer patients a second opinion free of charge *via* a reference figure for this purpose, while only four regions have a call center for the regional cancer network. Given participants’ emphasis on the importance of a highly effective cancer network for meeting oncology health targets, the sharing of patient data on digital platforms and the use of telemedicine applications would be of tremendous value.

##### 3.3.1.7 Domain: budget increase

“As is” – See explanation above (3.3.1.3)

“To be” scenario - In light of the future improvements in patient survival and quality of life (new treatments, creation of care pathways), institutions should plan to increase/redistribute the budget allocated to the management of this disease, taking the possible savings generated by an improvement in the efficiency of the patient management model into account.

The potential for more advanced therapies in future may permit a more efficient overall management and fewer expensive surgical procedures compared to the current economic model of the disease. Recommendations for a more efficient use of economic resources might include the delisting of treatments which are no longer used, supply contracts for new drugs based on expected outcomes (pay-for-performance), substitution of originator cancer medicines with biosimilars as these become available (to free up resources), as well as targeted and more appropriate treatment indications.

## 4 Discussion

Based on the results of this multidimensional consensus, the expert panel agreed in the end to indicate the following areas and gaps that might be more actionable in the future, recommending several actions to be proposed to the various stakeholder of the Italian healthcare system.

### 4.1 Survival, diagnosis and awareness

Participants stressed that this disease currently has a high probability of progression, making it difficult to cure, and underlined the importance of screening asymptomatic members of the public to diagnose the disease in its early stages. There is currently a lack of scientific evidence to support screening programs for urothelial cancer among the general public and high risk groups (unspecified primary prevention), as shown by the poor performance of current diagnostic tests and their failure to reduce mortality.

The panel considered biomarkers to be the future of scientific research to develop non invasive diagnostic strategies for the detection of urothelial cancer at its early stages. On this topic, participants debated who should be most responsible for promoting clinical research and attracting economic resources for this objective, including from independent sources, concluding that the institutions should establish a virtuous process for this purpose.

With regard to education about the disease to shorten diagnostic times, participants felt it important to raise awareness of the disease not only among the general public, but also among general practitioners, often the patient’s first point of contact when symptoms begin. Participants also recommended an improvement in communication between general practitioners and other specialists to improve the early recognition of symptoms. Participants also stressed the need for effective health campaigns aimed at different target audiences, with the involvement of the institutions (in the broadest sense), scientific societies and patient associations, with promotion of the latter two as valuable members of the care process.

Finally, participants mentioned the need to introduce specific training programs tailored to the various healthcare professionals involved in the care pathways.

### 4.2 Treatment, MDT, molecular tumor board and community healthcare services

Despite the recent improvement in available treatment options, improving personalization of the care pathway will require increasing attention to patient selection, a priority in the light of imminent new target therapies for locally advanced/metastatic urothelial cancer which will permit the creation of more targeted treatment pathways, leading to increased survival and quality of life. While this shift in focus will require greater resources, these will be offset by a redistribution of the savings resulting from the streamlining of the patient management model (with the hospital pharmacist an important figure in this process), and early patient management by a multidisciplinary team is a priority for guaranteeing access to all the available treatment options. Consisting of a variety of healthcare professionals, the MDT needs to offer a multidisciplinary approach which cares for the patient from the outset. The patient should be taken on by the MDT as quickly as resources allow, while the degree of care should be tailored to their individual needs. While MDTs permit an effective management of all aspects of patient care, they also require a management of the resulting organizational implications by healthcare trusts and regional healthcare systems. Individual MDTs must therefore be developed on the basis of existing community healthcare services and the most appropriate organizational models (regional cancer networks, each with their own model). Moreover, in the era of precision medicine, a single or bi-weekly, one-hour MTB meeting are highly recommended, to review patient potentially selected to targeted therapies, based on tissue biomarkers. The immunohistochemical (IHC), *in situ* hybridization (FISH) and next generation sequencing (NGS) analyses, are actually comprehensive methods available in routine practices, useful for pre-screen genomic alterations. The single named biomarker, the methods (IHC, FISH or NGS) and the clinical relevance are modern molecular triage opening discussion for patient selection at MTBs. The online portal is the repository necessary for immediate integrative reports and data from other genomic laboratories testing tissue or liquid biopsy samples may also provide additional information. Finally, actionable, complicated, or associated with novel treatment decisions not fully incorporated into clinical practice need discussion simultaneously by the MDT and MTB (67). MTB may provide recommendations for clinical care to the MDT within different categories: standard therapy and clinical trials, off-label therapies, germline testing and subsequent genetic counseling and advice for classifying tumor where origin has not been defined (67).

### 4.3 Holistic approach to the patient and caregiver, care pathways, quality of life

The majority of patient care currently takes place in hospital, with the generation of indirect costs (loss of productivity) which are also borne by caregivers. Participants therefore stressed the need to review community healthcare services, and the criteria for and coverage under the current exemption system in Italy, which exempts patients with specific health conditions from the payment of fees for certain drugs, tests and medical treatments. These factors can affect survival and quality of life, and trigger a phenomenon known as financial toxicity, a term coined to describe the harmful effects of high treatment costs on cancer patients and their caregivers.

In organizational terms, participants stressed the need for all regional health services to create a care pathway (highlighting that only two of the twenty-one Italian regions currently have an approved care pathway for this cancer), and that management of patients by all healthcare professionals in the care pathway for this disease is an objective yet to be met.

Participants also discussed the need to involve patient representatives (those best placed to judge the times when patients are most vulnerable), hospital pharmacists and GPs in the process of care pathway definition, thus also offering them important training opportunities.

A topic widely discussed was the impact of the disease on quality of life, highlighting how the physical, psychological and social effects of the disease are not given sufficient consideration in the current model of care, and the need for a much greater focus on these aspects, which can hinder the patient’s adherence to treatment and their response to it.

While underlining the need to implement patient support programs as soon as possible *via* an initial psychological screening process where appropriate, participants discussed the need for a language and a strategy that is as uniform as possible across the various services, and telemedicine may constitute a useful tool in this respect. To support caregivers, participants also highlighted the need to promote opportunities for meetings between caregivers, since they share similar social, psychological, employment, and family issues.

### 4.4 Improving the efficiency of expenditure and patient management

Regarding the economic model and the possibility of making it more efficient, participants shared the view that treating patients with increasingly expensive drugs will translate to higher costs, and that there are ample margins for increasing efficiency during the initial stage of the disease.

With regard to minimum monitoring indicators, the panel recommended adopting those which have emerged from the work of MDTs for this cancer: minimum number of radical cystectomies per center per year; number of patients undergoing radical cystectomy who require hospitalization within 90 days of surgery due to post-operative complications per center/year; number of patients deceased due to causes correlated to radical cystectomy within 30 days of surgery per center/year; ratio between patients with severe perioperative complications (Clavien-Dindo grade IV), and total number of patients with post-operative complications per center/year.

As regards the implementation of each indicator in terms of their individual objectives, the panel recommends taking the differences between regional health services and the different speeds of implementation into account.

## 5 Conclusion

The current management and treatment model for advanced urothelial carcinoma offers very limited treatment options with poor tolerability, leading many patients to abandon treatment, with numerous relapses and poor outcomes.

Effective patient management is also hampered by a limited number of multidisciplinary teams and treatment pathways, both tools which permit an early and effective diagnosis and improved quality of life.

While innovative recent and upcoming promising new treatments (such as avelumab, enfortumab vedotin, erdafitinib and sacituzumab, pembrolizumab, nivolumab) will significantly change the clinician’s approach to patient treatment and quality of life, clinicians will inevitably be required to juggle this positive clinical impact with the economic implications of the new treatment options, raising issues of healthcare budget sustainability. Along with precision medicine, promising actionable, complicated, or associated with novel treatment decisions not fully incorporated into clinical practice need discussion simultaneously by the MDT and MTB. Again, MTBs may provide recommendations for clinical care to the MDT within different categories such as standard therapy and clinical trials, off-label therapies, germline testing and subsequent genetic counseling (67).

In this regard, the project offers a modular structure to be applicable up to local level, indicating a minimum level of acceptance: accuracy, adequate medical education for patients and caregivers, access to innovative therapeutical tools for a more efficient patient care (eg PDTA-Integrated Diagnostic and Therapeutic Pathway and relative indicators of quality).

In conclusion, this project set out to respond to the need to generate a consensus among the multiple players in the Italian healthcare ecosystem to ensure effective patient management and an optimal assimilation of treatment innovations, their value and the consequent economic impact in the near future and can be considered an innovative analysis model for other healthcare systems or countries: in this regard, the model has been applied to public and hybrid public-private healthcare systems, it is clear that the same methodology might produce different results once applied to very different healthcare systems.

## Data availability statement

The original contributions presented in the study are included in the article/supplementary material. Further inquiries can be directed to the corresponding author.

## Ethics statement

This study consisted of a report of expert opinions that are from the authors, who all have given consent to the use of their information and opinion for this manuscript. No patient data were collected, so no ethical approval was required to perform this study.

## Author contributions

Conceptualization, SB, GP, SR, and FR; methodology, SB, GP, SR, and FR; writing-original, SR, FR, and SB; literature search, FR; writing-review and editing, GP, GNC, PB, RI, ML, VB, PZ, AG, FM, ID’A, DM, EF, LM, FD, DCA, DCR, SE, GLC, FF, ES, AM, DL, FR, SR and SB; manuscript revision MB. All authors contributed to the article and approved the submitted version.

## References

[1] Ferlay J Soerjomataram I Ervik M Dikshit R Eser S Mathers C. GLOBOCAN 2012: Estimated Cancer Incidence, Mortality and Prevalence Worldwide in 2012. WHO (2012).

[2] AIOM-AIRTUM- fondazione AIOM, PASSI, SIAPEC-IAP, ONS. I numeri del cancro in italia 2021. Brescia: Intermedia Editore (2021).

[3] Loriot Y Necchi A Park SH Garcia-Donas J Huddart R Burgess E. Erdafitinib in locally advanced or metastatic urothelial carcinoma. N Engl J Med (2019) 381(4):338–48. doi: 10.1056/NEJMoa1817323 31340094

[4] Petrylak DP Powles T Rosenberg JE. Enfortumab vedotin in advanced urothelial carcinoma. N Engl J Med (2021) 385(1):93–4. doi: 10.1056/NEJMc2106975 34192441

[5] Witjes JA Babjuk M Bellmunt J Bruins HM De Reijke TM De Santis M. EAU-ESMO consensus statements on the management of advanced and variant bladder cancer–an international collaborative multistakeholder efforty. Eur Urol. (2020) 77(2):223–50. doi: 10.1016/j.eururo.2019.09.035 31753752

[6] Wood L Bjarnason GA Black PC Cagiannos I Heng DY Kapoor A. Using the Delphi technique to improve clinical outcomes through the development of quality indicators in renal cell carcinoma. J Oncol Pract (2013) 9(5):e262–7. doi: 10.1200/JOP.2012.000870 23943895

[7] Meshkat B Cowman S Gethin G Ryan K Wiley M Brick A. Using an e-Delphi technique in achieving consensus across disciplines for developing best practice in day surgery in Ireland. J Hosp Adm (2014) 3:1–8. doi: 10.5430/jha.v3n4p1

[8] Gustafson DH Shukla RK Delbecq A Walster GW. A comparative study of differences in subjective likelihood estimates made by individuals, interacting groups, Delphi groups, and nominal groups. Organ Behav Hum Perf (1973) 9:280–91. doi: 10.1016/0030-5073(73)90052-4

[9] Loblaw DA Prestrud AA Somerfield MR Oliver TK Brouwers MC Nam RK. American Society of clinical oncology clinical practice guidelines: formal systematic review-based consensus methodology. J Clin Oncol (2012) 30(25):3136–40. doi: 10.1200/JCO.2012.42.0489 22778311

[10] Bellmunt J Necchi A De Wit R Lee J-L Fong L Vogelzang NJ. Pembrolizumab (pembro) versus investigator’s choice of paclitaxel, docetaxel, or vinflunine in recurrent, advanced urothelial cancer (UC): 5-year follow-up from the phase 3 KEYNOTE-045 trial. JCO (2021) 39(15_suppl):4532–2. doi: 10.1200/JCO.2021.39.15_suppl.4532

[11] Merck Sharp & Dohme Corp. A phase III randomized clinical trial of pembrolizumab (MK-3475) versus paclitaxel, docetaxel or vinflunine in subjects with recurrent or progressive metastatic urothelial cancer. Available at: https://clinicaltrials.gov/ct2/show/NCT02256436.

[12] AIOM. Linee Guida, Tumori dell’urotelio. Edizione 2021, aggiornata ottobre (2021).

[13] Hepp Z Shah SN Smoyer K Vadagam P. Epidemiology and treatment patterns for locally advanced or metastatic urothelial carcinoma: a systematic literature review and gap analysis. J Manag Care Spec Pharm (2021) 27(2):240–55. doi: 10.18553/jmcp.2020.20285 PMC10394179 33355035

[14] De Nunzio C Giannatempo P Passalacqua R Fiorini E Luccarini I Brigido A. Epidemiology and unmet needs of bladder cancer in Italy: a critical review. Minerva Urol Nefrol. (2020) 72(1):1–12. doi: 10.23736/S0393-2249.19.03498-2 31692303

[15] Cumberbatch MGK Jubber I Black PC Esperto F Figueroa JD Kamat AM. Epidemiology of bladder cancer: a systematic review and contemporary update of risk factors in 2018. Eur Urol. (2018) 74(6):784–95. doi: 10.1016/j.eururo.2018.09.001 30268659

[16] National cancer institute surveillance, epidemiology, and end results program. Cancer Stat facts: bladder cancer. (2020).

[17] The white paper, 2015 F.A.V.O. systematic review of the evidence . European Partnership for Action Against Cancer (EPAAC. Available at: www.epaac.eu (Accessed 22 June 2018).

[18] Shirodkar SP Lokeshwar VB. Bladder tumor markers: from hematuria to molecular diagnostics–where do we stand? Expert Rev Anticancer Ther (2008) 8(7):1111–23. doi: 10.1586/14737140.8.7.1111 PMC5515477 18588456

[19] Westhoff E Maria de Oliveira-Neumayer J Aben KK Vrieling A Kiemeney LA. Low awareness of risk factors among bladder cancer survivors: new evidence and a literature overview. Eur J Cancer. (2016) 60:136–45. doi: 10.1016/j.ejca.2016.03.071 27125965

[20] Cumberbatch MG Rota M Catto JW La Vecchia C. The role of tobacco smoke in bladder and kidney carcinogenesis: a comparison of exposures and meta-analysis of incidence and mortality risks. Eur Urol. (2016) 70(3):458–66. doi: 10.1016/j.eururo.2015.06.042 26149669

[21] PDQ Screening and prevention editorial board. bladder and other urothelial cancers screening (PDQ®): health professional version. 2021 jun 29. In: PDQ Cancer information summaries. Bethesda (MD: National Cancer Institute (US. 26389217

[22] Witjes JA Bruins HM Cathomas R Compérat E Cowan NC Efstathiou JA. Guidelines associates: e. linares espinós, m. rouanne, y. neuzillet. EAU guidelines. edn. presented at the EAU annual congress Milan (2021). Arnhem, The Netherlands: EAU Guidelines Office. Available at: https://uroweb.org/guideline/bladder-cancer-muscle-invasive-and-metastatic/

[23] National Collaborating Centre for Cancer (UK). Bladder Cancer: Diagnosis and Management. London: National Institute for Health and Care Excellence (NICE); (2015).

[24] Zlotta AR Roumeguere T Kuk C Alkhateeb S Rorive S Lemy A. Select screening in a specific high-risk population of patients suggests a stage migration toward detection of non-muscle-invasive bladder cancer. Eur Urol (2011) 59(6):1026–31. doi: 10.1016/j.eururo.2011.03.027 21458152

[25] Ng K Stenzl A Sharma A Vasdev N. Urinary biomarkers in bladder cancer: a review of the current landscape and future directions. Urol Oncol (2021) 39(1):41–51. doi: 10.1016/j.urolonc.2020.08.016 32919875

[26] Krogsbøll LT Jørgensen KJ Gøtzsche PC. Screening with urinary dipsticks for reducing morbidity and mortality. Cochrane Database Syst Rev (2015) 1(1):CD010007. doi: 10.1002/14651858.CD010007.pub2 25626128 PMC8928469

[27] Bossi A Di Lallo A Hurle R Mandressi A Migliari R Pappagallo GL. Linee guida per il Carcinoma Vescicale: 1.il tumore superficiale. AURO.it Associazione Urologi Ospedalieri (2001), 34–6 Available at: https://www.auro.it/linee-guida/05_CARCINOMA%20VESCICALE%201%20-%20Il%20tumore%20superficiale.pdf

[28] ESMO Congress Powles T. (2021).

[29] Cancer Genome Atlas Research Network. Comprehensive molecular characterization of urothelial bladder carcinoma. Nature (2014) 507(7492):315–22. doi: 10.1038/nature12965 PMC3962515 24476821

[30] Robertson AG Kim J Al-Ahmadie H Bellmunt J Guo G Cherniack AD. Comprehensive molecular characterization of urothelial bladder carcinoma. Cell (2017) 171(3):540–556.e25. doi: 10.1016/j.cell.2017.09.007 PMC5687509 28988769

[31] Powles T Assaf ZJ Davarpanah N Hussain M Oudard S Gschwend JE. 1O Clinical outcomes in post-operative ctDNA-positive muscle-invasive urothelial carcinoma (MIUC) patients after atezolizumab adjuvant therapy. Ann Oncol (2020) 31(suppl. 7):S1417–24. doi: 10.1016/j.annonc.2020.10.486

[32] EAU Congress (2021).

[33] Lee HW. H.K. Fibroblast Growth Factor Inhibitors for Treating Locally Advanced/Metastatic Bladder Urothelial Carcinomas via Dual Targeting of Tumor-Specific Oncogenic Signaling and the Tumor Immune Microenvironment. Int J Mol Sci (2021) 22:9526. doi: 10.3390/ijms22179526 34502435 PMC8431699

[34] Rosenberg JE Flaig TW Friedlander TW Milowsky MI Srinivas S Petrylak DP. Study EV-103: Preliminary durability results of enfortumab vedotin plus pembrolizumab for locally advanced or metastatic urothelial carcinoma. J Clin Oncol (2020) 38(6_suppl):441.

[35] Yu EY Petrylak DP O'Donnell PH Lee JL van der Heijden MS Loriot Y. Enfortumab vedotin after PD-1 or PD-L1 inhibitors in cisplatin-ineligible patients with advanced urothelial carcinoma (EV−201): a multicentre, single-arm, phase 2 trial. Lancet Oncol (2021) 22(6):872–82. doi: 10.1016/S1470-2045(21)00094-2 33991512

[36] Petrylak DP Flaig TW Mar N Gourdin TS Srinivas S Rosenberg JE. Study EV-103 cohort h: antitumor activity of neoadjuvant treatment with enfortumab vedotin monotherapy in patients (pts) with muscle invasive bladder cancer (MIBC) who are cisplatin-ineligible. J Clin Oncol (2022) 40(suppl 6):435. doi: 10.1200/JCO.2022.40.6_suppl.435

[37] Tagawa ST Balar AV Petrylak DP Kalebasty AR Loriot Y Fléchon A. TROPHY-U-01: a phase II open-label study of sacituzumab govitecan in patients with metastatic urothelial carcinoma progressing after platinum-based chemotherapy and checkpoint inhibitors. J Clin Oncol (2021) 39(22):2474–85. doi: 10.1200/JCO.20.03489 PMC8315301 33929895

[38] Jelenc M Van Hoof E Albreht T Meglič M Seljak M Krnel SR. Joint action European partnership for action against cancer. Arch Publ Health (2012) 70(1):24. doi: 10.1186/0778-7367-70-24 PMC3542586 23095375

[39] Communication from the commission on action against cancer: European partnership. COM (2009).

[40] Prades J van Hoof E Remue E and Borras JM. Multidisciplinary teams in cancer care: a systematic review of the evidence. deliverable 1, specific objective 1.1; work package 7 of the European partnership for action against cancer (EPAAC). Available at: www.epaac.eu (Accessed 22 June 2018).

[41] Fennell ML Das IP Clauser S Petrelli N and Salner A. The organization of multidisciplinary care teams: modeling internal and external influences on cancer care quality. J Natl Cancer Inst Monogr (2010) 2010(40):72–80. doi: 10.1093/jncimonographs/lgq010 PMC3482953 20386055

[42] Kurpad R Kim W Rathmell WK Godley P Whang Y Fielding J. A multidisciplinary approach to the management of urologic malignancies: does it influence diagnostic and treatment decisions? Urol Oncol (2011) 29(4):378–82. doi: 10.1016/j.urolonc.2009.04.008 19576797

[43] AIOM, AIRB, AIRO, AUrO, CIPOMO, SIU, SIUrO. (2017). Il Team Multidisciplinare Uro-Oncologico Una Sfida Comune. Consensus Conference.

[44] European Partnership action against cancer consensus group. policy statement on multidisciplinary cancer care. Eur J Cancer (2014).

[45] Rapporto quarta indagine nazionale sullo stato di attuazione delle R.O.R. Agenas (2021). Available at: https://www.agenas.gov.it/.

[46] 13° rapporto sulla condizione assistenziale dei malati oncologici, F.A.V.O (2021). Available at: https://osservatorio.favo.it/tredicesimo-rapporto/.

[47] Report aggiornamento delle R.O.R. Agenas (2022). Available at: https://www.agenas.gov.it/

[48] Mohamed NE Pisipati S Lee CT Goltz HH Latini DM Gilbert FS. Unmet informational and supportive care needs of patients following cystectomy for bladder cancer based on age, sex, and treatment choices. Urol Oncol (2016) 34(12):531.e7–531.e14. doi: 10.1016/j.urolonc.2016.06.010 27449687

[49] Benner C Greenberg M Shepard N Meng MV Rabow MW. The natural history of symptoms and distress in patients and families following cystectomy for treatment of muscle invasive bladder cancer. J Urol. (2014) 191(4):937–42. doi: 10.1016/j.juro.2013.10.101 24184369

[50] PDTA del tumore della vescica; associazionepalinuro.com/pdta-del-tumore-alla-vescica (2019). Available at: https://www.associazionepalinuro.com/cosa-facciamo/area-scientifica/pdta-del-tumore-alla-vescica.html

[51] Bergerot CD Philip EJ Bergerot PG Pal SK. Distress and quality of life among patients with advanced genitourinary cancers. Eur Urol Focus. (2020) 6(6):1150–4. doi: 10.1016/j.euf.2019.10.014 31711933

[52] Cancro della vescica: una guida per il paziente 2016 – linee guida per la pratica cinica ESMO (2016). Available at: https://www.esmo.org.

[53] Pham H Torres H Sharma P. Mental health implications in bladder cancer patients: a review. Urol Oncol (2019) 37(2):97–107. doi: 10.1016/j.urolonc.2018.12.006 30584034

[54] Graffigna G Barello S. Engagement: un nuovo modello di partecipazione in sanità. Il Pensiero Scientifico Editore (2017).

[55] PDTA urologico gruppo interdisciplinare cure – tumore vescicale infiltrante. Azienda Ospedaliera Ordine Mauriziano – Torino, Piemonte (2014).

[56] PDTA in urologia oncologica – tumore vescicale e prostatico. Azienda Sanitaria Provinciale 1 di Agrigento, Sicilia (2014).

[57] PDTA gruppo interdisciplinare cure – tumore vescicale. Azienda Sanitaria Locale Torino 3, Torino, Piemonte (2016).

[58] PDTA tumore vescicale e del tratto urinario superiore Azienda ospedaliera universitaria città della salute e della scienza, torino, piemonte (2016). Available at: https://www.cittadellasalute.to.it/index.php?option=com_content&view=article&id=14673:vescica-e-alta-via-escretrice&catid=409:pdt-oncologici-molinette&Itemid=615

[59] PDTA urologico - gruppo interdisciplinare cure – tumore vescicale. Azienda Sanitaria Locale Verbano-Cusio-Ossola, Verbania, Piemonte (2016).

[60] PDTA in oncologia uro-oncologica – tumore vescicale. Azienda Sanitaria Locale, Torino 4, Torino, Piemonte (2017).

[61] PDTA per pazienti affetti da tumori della vescica - rete oncologica veneta (2018). Available at: https://salute.regione.veneto.it/c/document_library/get_file?uuid=cd02da8d-e266-4194-ae99-ad2c1c2881d0&groupId=534936

[62] PDTA per il tumore della vescica - rete oncologica campana (2020). Available at: https://www.reteoncologicacampana.it/wp-content/uploads/2020/06/PDTA-VESCICA_ALL-8.pdf

[63] Grimm T Grimm J Buchner A Schulz G Jokisch F Stief CG. Health-related quality of life after radical cystectomy and ileal orthotopic neobladder: effect of detailed continence outcomes. World J Urol. (2019) 37(11):2385–92. doi: 10.1007/s00345-019-02643-8 30701335

[64] Leal J Luengo-Fernandez R Sullivan R Witjes JA. Economic burden of bladder cancer across the European union. Eur Urol (2016) 69(3):438–47. doi: 10.1016/j.eururo.2015.10.024 26508308

[65] Gerace C Montorsi F Tambaro R Cartenì G DeLuca S Tucci M. Cost of illness of urothelial bladder cancer in Italy. ClinicoEconomics Outcomes Res (2017) 9:433–42. doi: 10.2147/CEOR.S135065 PMC5533568 28769578

[66] Perrone F Gallo C. Le dolenti note. la tossicità finanziaria del paziente oncologico. Recenti Progr.Med. (2016) 107:619–21. 10.1701/2502.26224 27997003

[67] VanderWalde A Grothey A Vaena D Vidal G ElNaggar A Bufalino G. Establishment of a molecular tumor board (MTB) and uptake of recommendations in a community setting. J Pers. Med (2020) 10:252. doi: 10.3390/jpm10040252 33260805 PMC7711773

